# Supplementary material for: Are Faecal Microbiota Analyses on Species-Level Suitable Clinical Biomarkers? A Pilot Study in Subjects with Morbid Obesity
Source: Microorganisms. 2021 Mar 23;9(3):664. doi: 10.3390/microorganisms9030664 (PMC8005088; doi:10.3390/microorganisms9030664)
Supplement: Supplementary file 1 [file microorganisms-09-00664-s001.pdf]

microorganisms

Supplementary table S1: The 104 species targeted by PMP™ panels. The PMP™ qPCR probes are specific for the commercially available DMSZ and ATCC bacteria species genomes listed, and standard curves are made for each reference genome to enable quantification of the bacteria species.

Organism	BIO-ME assay number	Reference DNA	Standard curve coefficients	
			a	*b*
Acidaminococcus intestini	001	DSMZ 21505	-1.05061048	30.97420279
Akkermansia muciniphila	002	DSMZ 22959	-0.99200069	27.99878359
Alistipes finegoldii	003	DSMZ 17242	-0.98701559	30.05656981
Alistipes onderdonkii	004	DSMZ 19147	-1.04163499	30.7075846
Alistipes putredinis	005	DSMZ 17216	-1.09219549	31.50681812
Alistipes shahii	006	DSMZ 19121	-1.05918421	30.26379342
Anaerobutyricum hallii	007	DSMZ 3353	-0.94450275	30.52602934
Anaerostipes hadrus	008	DSMZ 3319	-0.9929078	30.08062728
Bacteroides caccae	009	DSMZ 19024	-0.94871781	29.78049382
Bacteroides cellulosilyticus	010	DSMZ 14838	-1.01554703	30.26067387
Bacteroides coprocola	011	DSMZ 17136	-1.03990522	30.12786534
Bacteroides dorei	012	DSMZ 17855	-0.915311	28.32832543
Bacteroides eggerthii	013	DSMZ 20697	-0.97839003	28.12306779
Bacteroides finegoldii	014	DSMZ 17565	-0.95839557	29.34717734
Bacteroides fragilis	015	DSMZ 2151	-1.04885914	30.02264856
Bacteroides intestinalis	016	DSMZ 17393	-1.01698864	29.74175488
Bacteroides massiliensis	017	DSMZ 17679	-0.98004837	28.272182
Bacteroides nordii	018	DSMZ 18764	-1.09923573	30.93153838
Bacteroides ovatus	019	DSMZ 1896	-1.02943587	30.55452496
Bacteroides plebeius	020	DSMZ 17135	-1.0586166	30.28596137
Bacteroides pyogenes	021	DSMZ 20611	-1.27181603	33.48445974
Bacteroides stercorirosoris	022	DSMZ 26884	-0.96905696	28.56165362
Bacteroides stercoris	023	DSMZ 19555	-1.04436195	30.52559276
Bacteroides thetaiotaomicron	024	DSMZ 2079	-0.99588526	29.97868842
Bacteroides vulgatus	025	DSMZ 1447	-0.99375166	30.04611268
Bacteroides xylanisolvens	026	DSMZ 18836	-1.04977123	30.2109513
Barnesiella intestinihominis	027	DSMZ 21032	-0.99423239	29.2892562
Bifidobacterium adolescentis	028	DSMZ 20083	-1.01681283	27.59507437
Bifidobacterium angulatum	029	DSMZ 20098	-0.97819653	29.71230634
Bifidobacterium bifidum	030	DSMZ 20456	-0.96510907	30.14128241
Bifidobacterium catenulatum	031	DSMZ 16992	-0.96028633	28.05646253
Bifidobacterium longum	032	DSMZ 20088, DSMZ 20219	-0.97281345	28.90702869
Bifidobacterium pseudocatenulatum	033	DSMZ 20438	-0.99173488	26.5329584
Bilophila wadsworthia	034	DSMZ 11045	-1.01117429	29.55859266
Blautia hydrogenotrophica	035	DSMZ 10507	-1.00971335	30.8131316
Blautia wexlerae	036	DSMZ 19850	-1.11039423	30.59460761
Butyrivibrio crossotus	037	DSMZ 2876	-1.03971266	31.01461527
Christensenella minuta	038	DSMZ 22607	-0.91593106	26.26144741
Citrobacter koseri	039	DSMZ 4595	-1.10189398	31.33504815

Clostridium bolteae	040	DSMZ 15670	-1.00074079	29.47113956
Clostridium butyricum	041	DSMZ 10702	-1.06325494	30.86244565
Clostridium citroniae	042	DSMZ 19261	-1.03889555	30.32929622
Clostridium leptum	043	DSMZ 753	-0.97427548	26.94196736
Clostridium nexile	044	DSMZ 1787	-1.07268777	30.80280579
Clostridium scindens	045	DSMZ 5676	-0.99249464	27.84473891
Clostridium symbiosum	046	DSMZ 934	-1.0426042	30.29530036
Collinsella aerofaciens	047	DSMZ 3979	-0.99547011	29.80091144
Collinsella intestinalis	048	DSMZ 13280	-1.09303418	30.50259202
Coprococcus catus	049	ATCC 27761	-1.08774726	31.09799167
Coprococcus comes	050	ATCC 27758	-0.97342159	28.83727187
Desulfovibrio piger	051	DSMZ 749	-1.06973085	30.06719604
Dorea formicigenerans	052	DSMZ 3992	-0.99322225	29.6728366
Dorea longicatena	053	DSMZ 13814	-1.00182917	30.09620124
Eggerthella lenta	054	DSMZ 2243	-1.06605138	30.99166682
Eisenbergiella massiliensis	055	DSMZ 101499	-1.04924994	30.17645471
Eisenbergiella tayi	056	DSMZ 26961	-1.08357349	29.91507109
Enterococcus dispar	057	DSMZ 6630	-1.07038602	30.14395012
Enterococcus faecalis	058	DSMZ 20478	-1.13299593	31.09974288
Enterococcus faecium	059	DSMZ 20477	-1.06061417	30.69427997
Enterococcus hirae	060	DSMZ 20160	-1.08260705	31.15199318
Erysipelatoclostridium ramosum	061	DSMZ 1402	-1.03375451	30.21368043
Escherichia coli	062	DSMZ 30083	-0.97357413	29.26532652
Eubacterium eligens	063	DSMZ 3376	-0.98195148	30.41186476
Eubacterium rectale	064	DSMZ 17629	-0.99768899	27.90880377
Eubacterium siraeum	065	DSMZ 15702	-0.99046026	29.86540549
Eubacterium ventriosum	066	DSMZ 3988	-0.94953202	29.78624085
Faecalibacterium prausnitzii	067	DSMZ 17677	-0.99727617	30.26448204
Fusobacterium varium	068	DSMZ 19868	-1.02620668	31.05126535
Haemophilus parainfluenzae	069	DSMZ 8978	-1.05366826	29.73433083
Hafnia alvei	070	DSMZ 30163	-1.06905313	30.0326339
Klebsiella variicola	071	DSMZ 15968	-0.96734124	29.58339885
Lactobacillus acidophilus	072	DSMZ 20079	-1.01748389	30.54203154
Lactobacillus animalis	073	DSMZ 20602	-1.06402298	30.18071147
Lactobacillus brevis	074	DSMZ 20054	-1.07579118	31.07036533
Lactobacillus jensenii	075	DSMZ 20557	-1.01853873	30.25214011
Lactobacillus paracasei	076	DSMZ 5622	-1.02335	31.48621617
Lactobacillus rhamnosus	077	DSMZ 20021	-1.09411101	30.21476944
Lactobacillus ruminis	078	DSMZ 20403	-0.98212992	29.68251303
Lactobacillus salivarius	079	DSMZ 20555	-1.03981024	29.71281834
Methanobrevibacter smithii	080	DSMZ 861	-1.02710283	29.23576913
Morganella morganii	081	DSMZ 30164	-1.06757616	29.66653129
Mycoplasma hominis	082	DSMZ 25592	-1.05382172	31.24076181
Odoribacter splanchnicus	083	DSMZ 20712	-1.06846044	30.45616819
Parabacteroides distasonis	084	DSMZ 20701	-0.98009259	28.75145783
Parabacteroides goldsteinii	085	DSMZ 19448	-1.05714489	30.39481878
Parabacteroides gordonii	086	DSMZ 23371	-0.98096663	29.21102888

Parabacteroides merdae	087	DSMZ 19495	-0.99997555	29.050045
Paraprevotella clara	088	DSMZ 19731	-1.03703379	30.52026965
Prevotella copri	089	DSMZ 18205	-0.98140764	28.8991566
Prevotella stercorea	090	DSMZ 18206	-1.12538489	31.84220512
Proteus mirabilis	091	DSMZ 4479	-1.05650952	31.0140791
Roseburia hominis	092	DSMZ 16839	-1.02223434	30.31886566
Roseburia intestinalis	093	DSMZ 14610	-1.04161979	27.450061
Roseburia inulinivorans	094	DSMZ 16841	-1.05804935	30.1451495
Ruminococcus albus	095	DSMZ 20455	-1.14233963	30.23344
Ruminococcus bromii	096	ATCC 27255	-1.00316784	29.49455476
Ruminococcus gnavus	097	DSMZ 108212	-1.00381811	29.66311384
Ruminococcus torques	098	ATCC 27756	-1.05778764	31.54960136
Streptococcus sanguinis	099	DSMZ 20567	-1.02869686	28.80567265
Streptococcus thermophilus	100	DSMZ 20617	-0.97681106	29.89745222
Subdoligranulum variabile	101	DSMZ 15176	-0.98936484	29.29031483
Sutterella wadsworthensis	102	DSMZ 14016	-0.9647432	28.73388786
Turicibacter sanguinis	103	DSMZ 14220	-0.98675327	30.71085981
Veillonella atypica	104	DSMZ 20739	-1.02831246	30.39838561
